# Integrating sol-gel and carbon dots chemistry for the fabrication of fluorescent hybrid organic-inorganic films

**DOI:** 10.1038/s41598-020-61517-x

**Published:** 2020-03-16

**Authors:** Stefania Mura, Róbert Ludmerczki, Luigi Stagi, Sebastiano Garroni, Carlo Maria Carbonaro, Pier Carlo Ricci, Maria Francesca Casula, Luca Malfatti, Plinio Innocenzi

**Affiliations:** 1 Laboratory of Materials Science and Nanotechnology, CR-INSTM, Department of Chemistry and Pharmacy, University of Sassari, Via Vienna 2, 07100 Sassari, Italy; 2 Department of Chemistry and Pharmacy, University of Sassari, Via Vienna 2, 07100 Sassari, Italy; 3 Department of Physics, University of Cagliari, Campus of Monserrato, sp n.8, km 0.700, 09042 Monserrato, Italy; 4 DIMCM-Department of Mechanical, Chemical, and Materials Engineering INSTM and University of Cagliari Via Marengo 2, I, 09123 Cagliari, Italy

**Keywords:** Optical properties and devices, Organic-inorganic nanostructures

## Abstract

Highly fluorescent blue and green-emitting carbon dots have been designed to be integrated into sol-gel processing of hybrid organic-inorganic materials through surface modification with an organosilane, 3-(aminopropyl)triethoxysilane (APTES). The carbon dots have been synthesised using citric acid and urea as precursors; the intense fluorescence exhibited by the nanoparticles, among the highest reported in the scientific literature, has been stabilised against quenching by APTES. When the modification is carried out in an aqueous solution, it leads to the formation of silica around the C-dots and an increase of luminescence, but also to the formation of large clusters which do not allow the deposition of optically transparent films. On the contrary, when the C-dots are modified in ethanol, the APTES improves the stability in the precursor sol even if any passivating thin silica shell does not form. Hybrid films containing APTES-functionalized C-dots are transparent with no traces of C-dots aggregation and show an intense luminescence in the blue and green range.

## Introduction

Carbon dots (C-dots) are fluorescent nanomaterials with optical properties comparable to semiconductor quantum dots. C-dots, however, have a much lower cost and environmental impact, which make them a hot topic of research^1,2^. A major advantage is the possibility to produce C-dots from an almost endless variety of precursors and methods. On the other hand, strict control of the properties through the process is still challenging to achieve, and the main efforts are now dedicated to obtaining reliable and reproducible synthesis.

The citric acid (CA) alone or in combination with other compounds is one of the most popular precursors for C-dots^3^. CA-based C-dots have on their surface different carboxy-groups, which increase the solubility and allow surface passivation or functionalization with organic molecules^4–6^ or polymers^7^. In general, pure CA C-dots, without any modification, show a weak emission, and doping^1^ with B, N, S, Si and P atoms is a possible solution to improve their quantum yield. Most of the CA C-dots are doped with nitrogen that enhances the luminescence by producing azo-compounds through the reaction between the carboxylic and amino groups; after carbonisation, they form water-dispersible and highly emitting C-dots^8^. Different amines have been used for this purpose, such as ethylenediamine (EDA)^2,5,9^, hexamethylenetetramine^6^, o-phenylenediamine (o-PD)^10^, triethylenetetramine^11^, hexadecylamine (HDA)^8^, and triethanolamine^6^. Quantum yields (QY) under 8% for most of the amines, with the exception of EDA^6^ which gives a QY of 86%, have been obtained. Urea, because of the high nitrogen content, can be used for doping CA C-dots^1,5,12–17^ and different methods have been developed so far. Hydrothermal treatment in an autoclave and microwave exposure are simple synthesis for producing luminescent C-dots from citric acid and urea. Low QY (16%)^1^ have been obtained by processing the dots via oven treatments, with even lower QY values observed in microwave processed samples (10–15%)^11,13,14^. An exception has been reported^15^ for a synthesis carried out in microwave. However the synthesis has been followed by a purification of the product through size exclusion chromatography achieving a QY of 73%. By using the autoclave treatment, one of the best QY has been obtained in toluene (51.2%), using rhodamine 6 G in ethanol as a standard^12^. higher value (78.8% QY) has been reported after an autoclave and annealing treatment at 250 °C, using quinine sulphate as reference standard^17^. All the recent works claim the difficulty in producing C-dots with high quantum yield (QY) and emissive products at higher wavelengths (>500 nm)^2^. In any case, a comparison of the different QY reported in literature should be made with care because most of the measures have been performed using a reference fluorescent dye (Relative Quantum Yield) and at different wavelengths; this could result in a much higher QYs with respect to the real absolute value.

Another important goal is the production of functionalized C-dots to be integrated into materials and processed in the form of films for solid-state photonic applications. C-dots usually exhibit a fair solubility in water and other polar solvents, however, the surface modification is expected to increase the solubility, avoiding the aggregation and improving the stability towards quenching. Up to now, only a few examples have reported on surface modification for embedding the C-dots into polymeric matrixes and even less in inorganic or hybrid organic-inorganic hosts obtained via sol-gel processing^9^. To fulfil this goal, we have developed highly emissive blue and green C-dots which have been functionalised by 3-aminopropyltriethoxysilane to achieve effective incorporation within a hybrid film. The post-synthesis grafting process, in fact, allows for a better control of the C-dots properties to be used for the design of sol-gel nanocomposites. Incorporation of the C-dots in a sol-gel material is, in fact, usually realised by dissolving the nanoparticles into the precursor sol^17–19^. This is the simplest route, but homogeneous dispersion of the C-dots within the film is difficult to achieve; at the same time, full integration with the sol-gel chemistry^20–22^ via sol-gel hydrolysis and condensation reactions^23^ is also hampered. In the present work, we have successfully obtained blue and green hybrid organic-inorganic films using C-dots modified with an organosilane which has allowed a full integration with the sol-gel chemistry. This step is very important for the future development of solid-state optical devices based on C-dots.

## Results and discussion

Keeping in mind the purpose of the present work we have developed a simple but effective synthesis which allows preparing highly fluorescent C-dots modified with an organofunctional alkoxide, 3-(aminopropyl)triethoxysilane (APTES). These C-dots can be well integrated into any sol-gel process to obtain fluorescent hybrid materials with controlled properties. We have prepared several batches of samples using different CA/urea molar ratios, and we have found that the best performances in the blue and green emission ranges are obtained using 1:2 (CU2) (blue) and 1:25 (CU25) (green) CA/urea molar ratios.

### Blue C-dots

**Figure 1:**
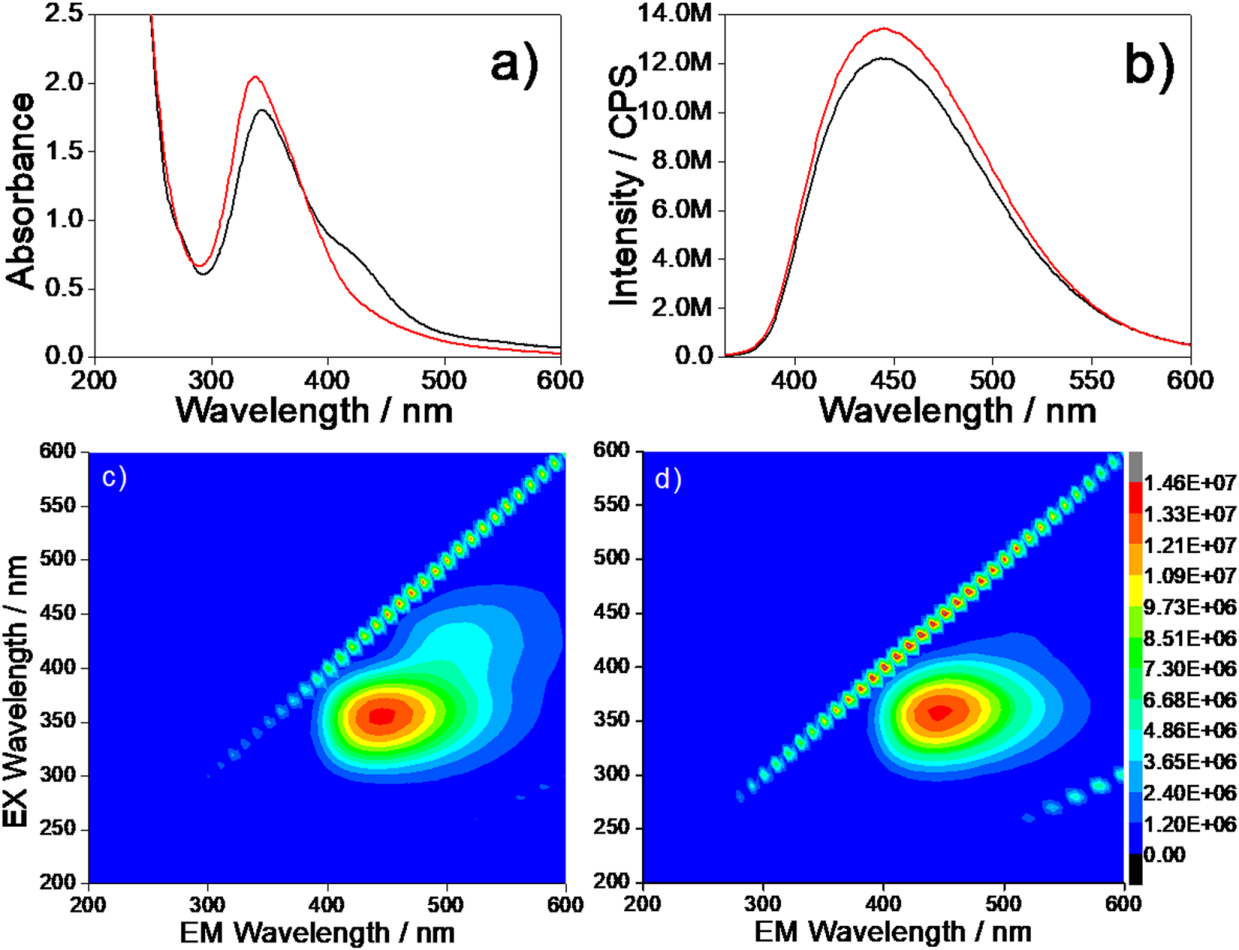
(**a**) UV-vis absorption spectra of CU2 aqueous solutions (concentration 0.1 mg mL^−1^) (black line), and after the functionalization with APTES (red line). (**b**) Emission spectra (λ_ex_ = 350 nm) of CU2 samples (1 mg L^−1^ concentration) (black line), and after the functionalization with APTES (red line). (**c**) 3D emission-excitation-intensity spectra of CU2 sample before and (**d**) after the functionalization with APTES in water.

The UV-Vis spectra of the CU2 sample dispersed in water (Fig. 1a) (black line) show two UV absorption bands attributed to the π- π* transition of aromatic sp^2^ domains (234 nm) and to the n - π* transition of C=O bond (345 nm)^13^. Another absorption band, peaking at 425 nm, is assigned to n- π* transitions of the functional groups on the C-dots surfaces.

Interestingly this band at longer wavelengths completely disappears upon functionalization of the CU2 dots with APTES in water (red line).

Figure 1c,d show the 3D photoluminescence spectra (excitation (y-axis), emission (x-axis), intensity (false colours scale)) of the C-dots samples in water before (Fig. 1c) and after the functionalization with APTES (Fig. 1d). The CU2 spectra are characterised by two main emissions, with maxima observed at 445 and 520 nm in emission and 350 and 420 nm in excitation, in accordance with the UV-vis spectra. The emission peaking at 445 nm (Fig. 1b) (λ_ex_ = 350 nm) increases in intensity after the functionalization with APTES while the 520 nm band in CU2 is no longer observed, such as in the UV-vis spectra (Fig. 1d). Because the UV-Vis absorption of citrazinic acid is characterised by a main band peaking around 340 nm, this molecule and its derivates should be the primary source of the fluorescence at around 450 nm^1,7,13^ (Supplementary Information, Fig. S1). The emission at 520 nm is, instead, quenched upon functionalization by APTES (*vide infra*).

The C-dots after the functionalization with APTES show a 12% increase of the absorbance, (Fig. 1a), a blue shift of the absorption maximum from 345 to 337 nm, and a 10% increase of emission intensity (Fig. 1b). We have also experimentally observed a significant change in the optical response if the reaction of APTES with the C-dots is carried out in ethanol or water (*vide infra*). In ethanol, in fact, the reactivity of APTES is slower and the solutions are more stable over time. If APTES is mixed with the C-dots in EtOH, the 425 nm band is not quenched anymore, an increase in absorbance and emission intensity are also observed (Supplementary Information, Fig. S2a,b).

The quantum yield (QY) of CU2, using quinine sulfate^24^ as reference (λ_ex_ = 365 nm, QY 55% in 0.1 M H_2_SO_4_) (Fig. S3), is 30% (Supplementary Information, Table S1). This is one of the highest values reported in the literature for blue C-dots synthesised by citric acid and urea.

### Green C-dots

To obtain green C-dots with a very high emission, we have increased the urea content up to CA: urea = 1: 25 molar ratio (CU25). This is an optimised value which gives highly fluorescent green C-dots.

**Figure 2:**
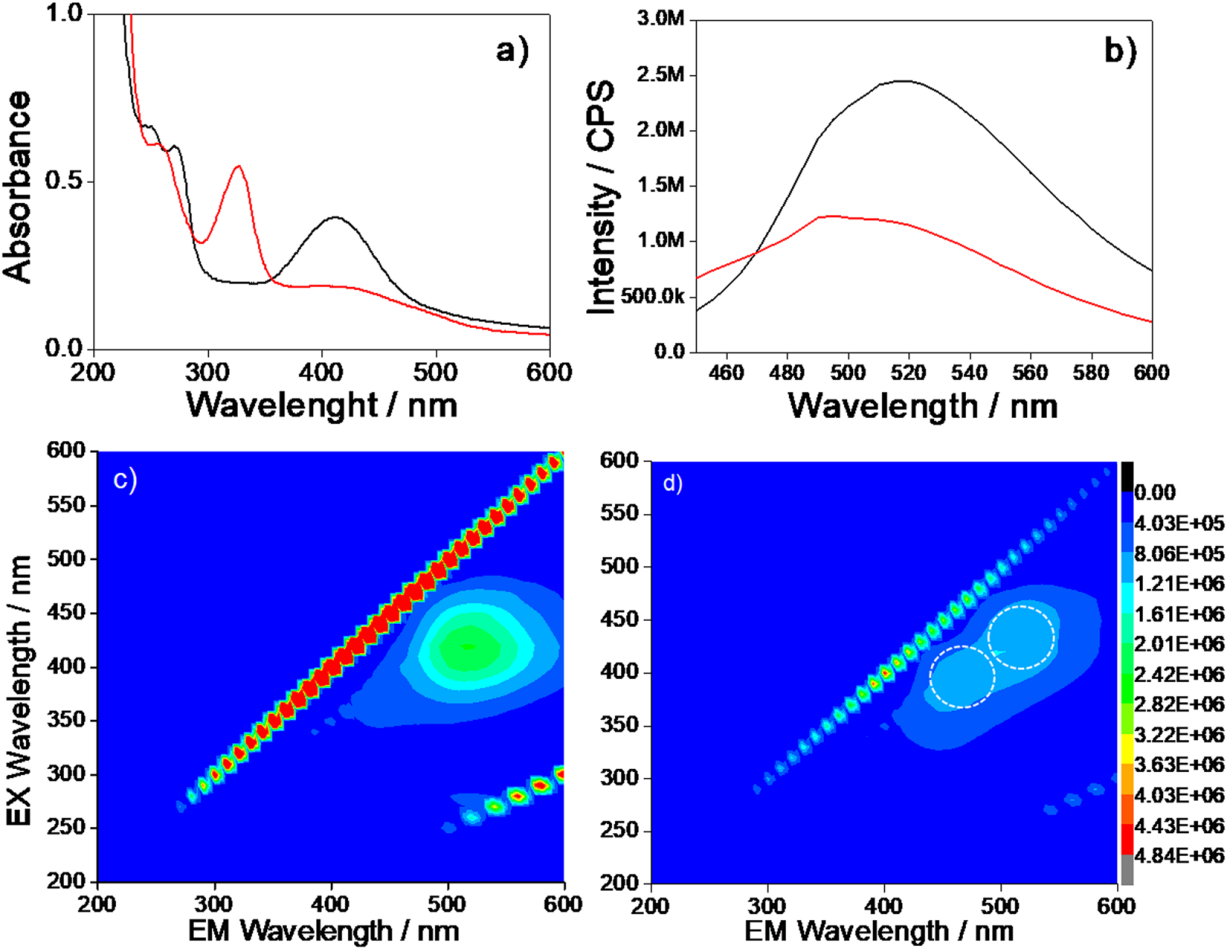
(**a**) UV-vis absorption spectra of CU25 C-dots in aqueous solutions (0.1 mg mL^−1^ concentration) (black line) and after the functionalization with APTES (red line). (**b**) Emission spectra (λ_ex_ = 400 nm) of CU25 C-dots (1 mg L^−1^ concentration), (black line) and after the functionalization with APTES (red line). 3D emission-excitation-intensity spectra (**c**) before and (**d**) after the functionalization with APTES.

The CU25 UV-vis spectra (Fig. 2a) show the presence of different absorption bands around 213, 248, 272 and 410 nm. In a recent article, Kasprzyk *et al*.^13^ have discussed the origin of the green emission of CA-urea C-dots formed at high molar ratios. They have identified 4-hydroxy-1H-pyrrolo[3,4-c]pyridine-1,3,6(2 H,5 H)-trione (HPPT), which forms from the citrazinic acid, as the main source of fluorescence. By comparing both the absorption and the photoluminescence of our samples with respect to those results, we attribute the green emission of CU2 and CU25 samples to the formation of HPPT fluorophores. The strong UV band gives a signature of this molecule at 410 nm attributed to n- π* transitions of the functional groups on the C-dots surfaces. The emission shows (λ_ex_ = 400 nm) a 70 nm shift of the maximum with respect to the blue C-dots, from 450 to 520 nm (Fig. 2b) and is 85% lower with respect to the blue C-dots. The absorption band at 410 nm after the APTES functionalization is quenched, while a new band at 328 nm is, instead, detected (Fig. 2a). The emission spectra (Fig. 2b) show the decrease in intensity of the band in the green (520 nm) and the rise of a 466 nm band in the blue (Fig. 2c,d). These effects are attributed, such as in the case of blue C-dots, to the formation of a silica passivation layer. Because the main source of green emission in the C-dots is mainly the surface^5^, the modification by APTES causes the changes in the absorption and emission spectra.

**Figure 3:**
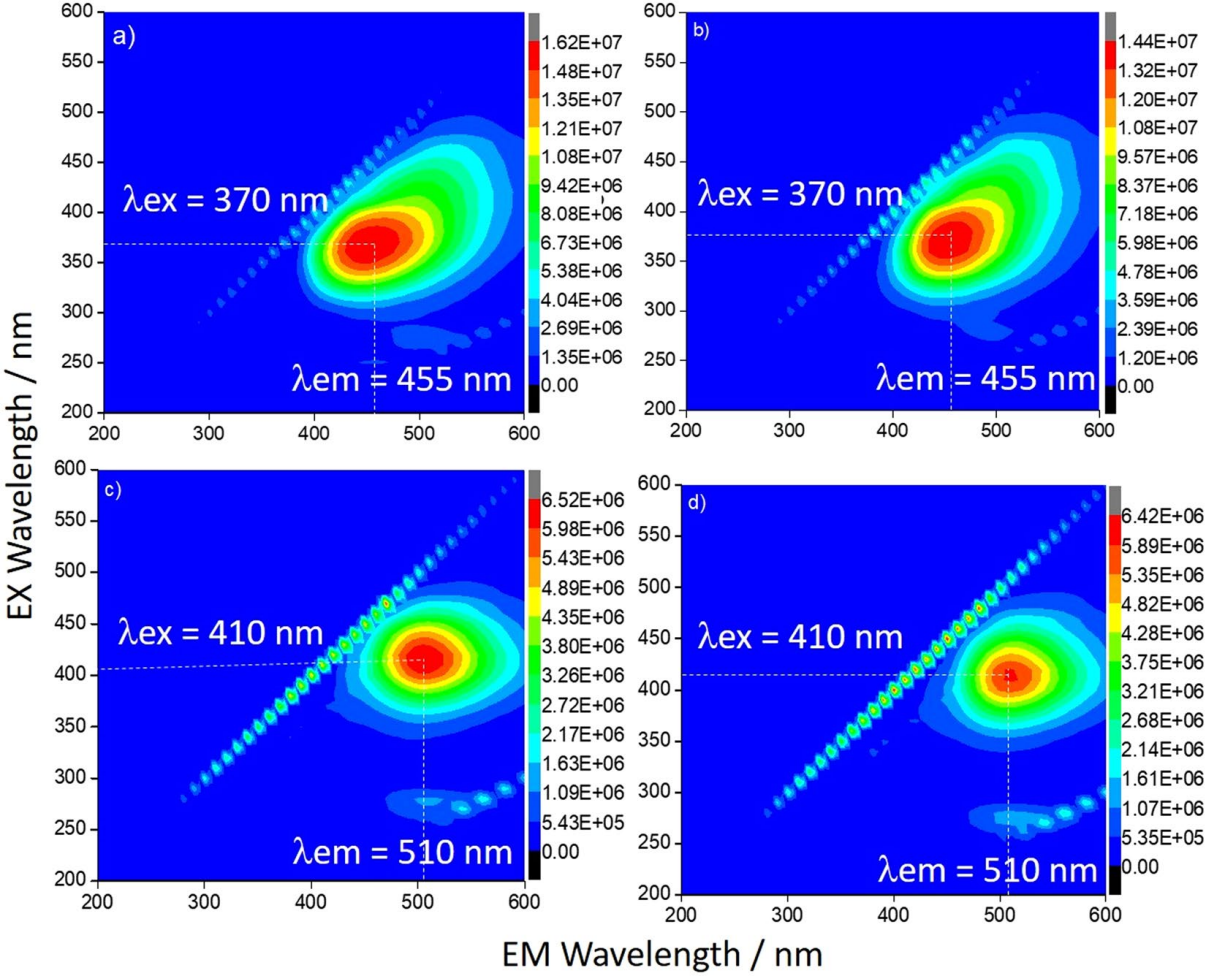
3D emission-excitation-intensity spectra of CU2 C-dots before (**a**) and after (**b**) the functionalization with APTES in ethanol. CU25 C-dots before (**c**) and after (**d**) the functionalization with APTES in ethanol (1 mg L^−1^ concentration). The dot lines in the spectra are a guide for eyes to identify the maxima of emission and excitation.

The UV-Vis and emission spectra (Supplementary Information, Fig. S4a,b) do not change if the C-dots are dissolved in ethanol, instead of water. The intensity of the spectra also results three times larger in comparison with the samples obtained by functionalization in water (Fig. 3). The PL emission spectra and digital images of CU2 and CU25 in water and EtOH have been directly compared in Fig. S5 for the sake of clarity. The CU2 dots show an asymmetric emission 3D map, which is composed of two components, by the UV-vis spectra. The CU25 spectra show, instead, differently from what has been observed in water (Fig. 3), only one component.

The CU25 C-dots in ethanol have shown a very high QY, 99.5% (Supplementary Information, Table S1), the highest to our knowledge reported in the literature for the method^23,24^ based on rhodamine 6 G (QY 95% in ethanol) as reference (Supplementary Information, Fig. S6).

We have also measured the Absolute Quantum Yield (AQY) using an integrating sphere. This value is not dependent on the reference standard (Relative Quantum Yield)^24^ and can be used to compare the QY of C-dots prepared in different experimental conditions. The AQY of blue C-dots is: 9.3% (CU2 in H_2_O), 29.1% (CU2 in EtOH) and 30.7% (CU25 in EtOH) and 12.12% (CU25 in H_2_O) (Supplementary Information, Fig. S7).

The difference between the relative and absolute QY of CU2 (30 and 29 respectively) and CU25 (99.5 and 30) is due to the dyes used as a standards for the relative QY estimate. In fact, these dyes show different QY depending on their purity, the solvent used, the concentration and the environmental conditions like the temperature. These differences can lead to a large variability of the relative quantum yield for the Rhodamine B and 6 G^24^. These QY values are lower than those obtained by a standard which gives in general an overestimation of the QY.

### TR-PL measurements

 The *time-resolved photoluminescence* (TR-PL) streak images have been collected by exciting four samples (CU2-APTES and CU25-APTES in water and ethanol) at 350, 370 and 400 nm in a time range of 100 ns, allowing to extract both the PL spectra and the decay time profiles.

**Figure 4:**
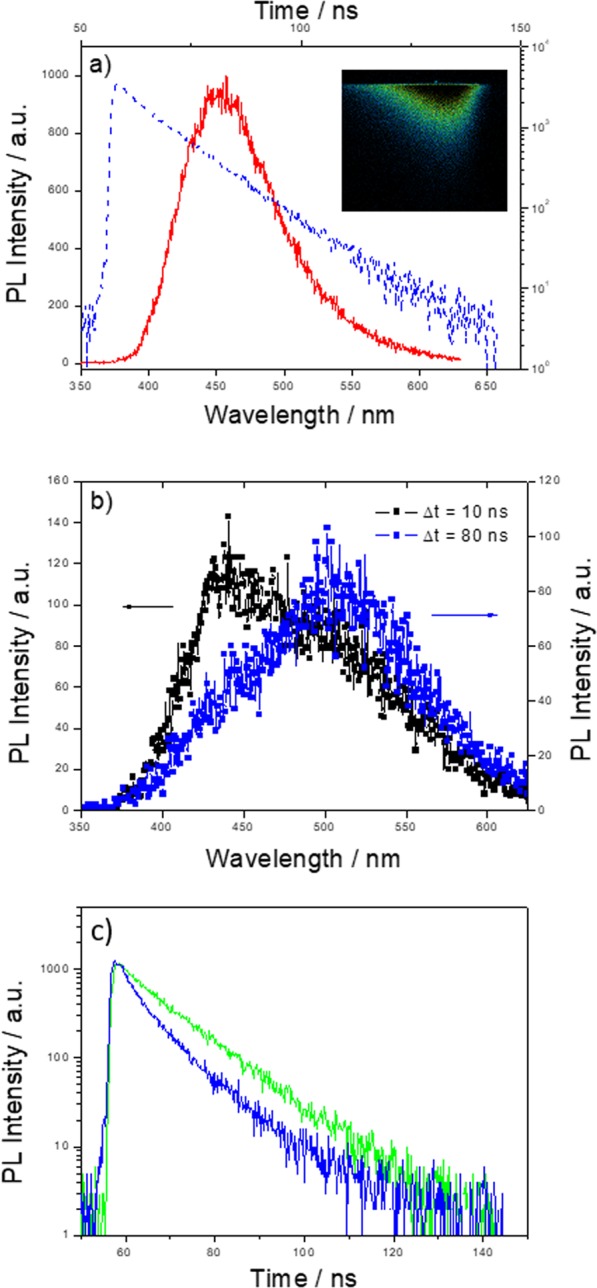
(**a**) Emission spectrum (red curve, bottom x-axis) and decay time profile (blue curve, top x-axis) of CU2 C-dots modified with APTES in water upon excitation at 350 nm. The inset shows the streak image. (**b**) Emission spectrum integrated over a time window of 10 ns (black curve) and of 80 ns (blue curve) of CU25 C-dots modified with APTES in ethanol excited at 350 nm. (**c**) Decay time profile of CU25 C-dots modified with APTES in water (blue curve) and in ethanol (green curve) excited at 350 nm and 400 nm respectively.

Figure 4a reports the case of CU2 C-dots modified with APTES in water as an example (the inset shows the streak image, time profile while the PL spectrum has been extracted by the integration of the full image over wavelength or time scale, respectively).

The whole set of samples has been analysed by fitting the time profiles with two exponential decays, of about 2–4 ns and 10 ns, with an estimated uncertainty of about 1.4 ns (estimated through the 10–90% rise time). The faster decay is beyond the object of the present investigation, being on the resolution limit of the experimental set-up for this time scale. We have checked it also over faster time windows (at 5 ns with an estimated uncertainty of 0.2 ns) confirming the value of about 2 ns. We have also observed that this decay affects the emission spectrum as a whole, suggesting the presence of a non-radiative contribution that can be accessed from the entire chromophore ensemble related to the recorded emission. The slower decay (≈10 ns) can be generally fitted in the energy space with two Gaussian bands picking around 2.5 eV (500 nm) and 2.8 eV (450 nm). The relative contribution of these bands depends on the synthesis conditions (blue or green C-dots), thus confirming the results gathered with the excitation/emission maps. We have not been able to single out the decay of each band, a feature indicating that the two emissions have comparable decays. To extract more information from the streak images, we have integrated the signal over different time windows, the first with a duration of 10 ns and the following 80 ns. The comparison of the two spectra allows assessing that the blue band is faster than the green one; its relative contribution being reduced in the second time windows (see Fig. 4b).

Then, considering the effect of APTES in water and ethanol in CU25 C-dots samples (Fig. 4c), we have compared the streak images excited at 350 and 400 nm for the water and ethanol cases, respectively. Indeed, in the first case we have been able to isolate a single emission band at about 450 nm with a decay time of 8.7 ns, while, in the second one, we have singled out the band at 510 nm with a decay time of 11.6 ns. For a better comparison, the lifetime of C-dots before and after the functionalization with APTES have been reported in Supplementary Information Table S2.

### The C-dots structure upon surface modification

**Figure 5:**
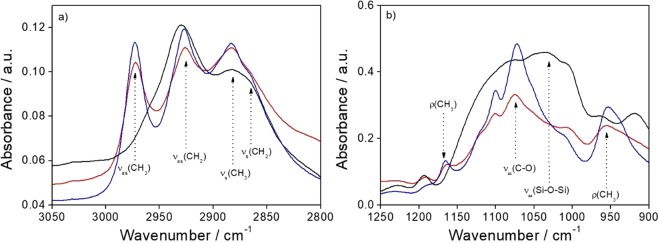
(**a**) FTIR absorption spectra in the 3050–2800 cm^−1^ range and (**b**) in the 1250–900 cm^−1^ range of the CU25 C-dots modified with APTES in water (black line) or in ethanol (red line); the APTES spectrum is also shown as a reference (blue line).

Figure 5a shows the FTIR absorption spectra in the 3050–2800 cm^−1^ range of the CU25 C-dots modified with APTES in water or ethanol; the APTES spectrum is also shown as a reference.

In this wavenumber range the stretching bands, symmetric and antisymmetric, of CH_2_ and CH_3_ are detected. The CH_3_ stretching modes (ν_as_ at 2974 cm^−1^ and ν_s_ at 2882 cm^−1^) are assigned to the alkoxy groups (–OCH_2_CH_3_) in APTES and the CH_2_ stretching modes (ν_as_ at 2926 cm^−1^ and ν_s_ at 2865 cm^−1^) to CH_2_ in the alkoxide and in the propyl chain in APTES. The spectra of the APTES modified C-dots using water or ethanol as solvent show a remarkable difference between them. The signal due to CH_3_ stretching in the alkoxy (ν_as_ at 2974 cm^−1^) completely disappears in the sample prepared using water as solvent. This indicates that APTES has reacted in hydrolytic condition and that the reaction has been autocatalysed by APTES whose addition in the aqueuos solution has increased the pH from neutral to basic (pH = 10). In ethanol, instead, APTES does not react, as shown by the FTIR spectra. Figure 5b shows the FTIR absorption spectra at lower wavenumbers, between 1250 and 900 cm^−1^. The spectra of APTES and CU25 C-dots functionalized with APTES in ethanol are characterised by the symmetric stretching ν_s_ (C-O) mode at 1073 cm^−1^ and the rocking ρ (CH_3_) mode at 1165 and 951 cm^−1^. The ρ(CH_3_) vibrational mode disappears in the sample functionalized with APTES in hydrolytic conditions, in accordance with Fig. 5a. This is an indication of the reaction of alkoxides groups which hydrolyse and form a silica structure. The CU25 sample modified with APTES in water shows the rise of a large band peaking around 1030 cm^−1^ with a shoulder around 1078 cm^−1^. These bands are the signature of formation of silica structures and are assigned to ν_as_ (Si-O-Si).

In principle, the C-dots surface carboxyls may react with the amines of APTES to form amide bonds. In general, this reaction is quite difficult to observe because the amines deprotonate the carboxylic acid and do not form reactive carboxylates. In the present conditions, the FTIR analysis (Supplementary Information, Fig. S8), has confirmed that a very small amount of amides are formed only when the CU25 dots react with APTES in water.

**Figure 6:**
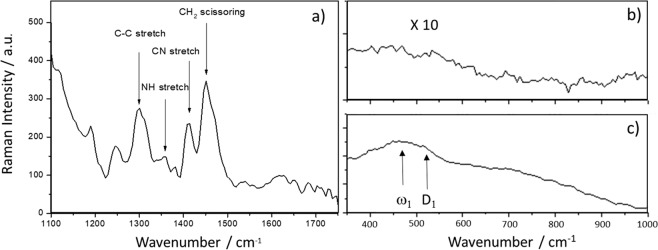
(**a**) Raman spectrum in the 1100–1750 cm^−1^ range of CU25-APTES in ethanol; Raman spectra in the 350–1000 cm^−1^ range of the CU25-APTES in (**b**) ethanol and (**c**) water.

Raman bands of CU25 C-dots modified with APTES in ethanol have been recorded in the spectral region between 1000 and 1700 cm^−1^ (Fig. 6a) where most of the bands can be assigned to the Raman spectrum of APTES molecules^25,26^. The main signal, at 1449 cm^−1^ is assigned to the CH_2_ scissoring mode^27^, while the scattering bands at 1307, 1349 and 1412 cm^−1^ are attributed to the stretching mode of C–C, N–H and CN, respectively^28^. The Raman spectrum of APTES, however, shows two additional bands between 1600 and 1650 cm^−1^ ^25^, related to the NH_2_ vibration modes (bending and stretching, respectively)^29^, that cannot be clearly observed in our spectra. Whilst no vibrational features of silica can be distinguished in the ethanol sample, the ω_1_ and D_1_ silica fingerprint bands, related to the five and sixfold ring structures and to fourfold ring structures respectively^30,31^, are clearly detected in the sample modified by APTES in water (Fig. 6b). These results confirm that, in ethanol, APTES does not react and do not form silica interconnected structures.

The analysis of FTIR and Raman data combined with the emission spectra of the samples suggests that the C-dots surface modification by APTES in hydrolytic conditions forms a thin silica passivating layer, which protects the C-dots from the emission quenching. The autocatalytic effect of APTES, which causes an increase of pH, determines the hydrolysis and condensation reactions of silica.

To get a better understanding of the effect of C-dots surface modification by APTES, a systematic analysis of the samples Z-potential has been performed. Table 1 lists the different values measured for CU2 and CU25 samples in water and ethanol without and with APTES. The samples are stable in water and ethanol, but after the addition of APTES in water, the Z-potential decreases in absolute value, indicating lower stability in solution. This is an effect of silica condensation, which causes quick precipitation of the C-dots. On the other hand, the C-dots/EtOH/APTES system keeps its stability, and the small decrease of the Z-potential is an indication of a small modification, probably via secondary bonding, of the particle’s surface. The surface modification by APTES does not affect the stability in solution, however it plays a primary role in increasing the fluorescence by avoiding the quenching effect due to the solvent molecules (*vide supra*).

**Table 1:** Zeta potential measurements of CU2 and CU25 samples before and after APTES modification.

	Water	Ethanol	Water + APTES	Ethanol + APTES
**CU2**	−28.0 mV	−27.8 mV	−6.2 mV	−21.5 mV
**CU25**	−38.8 mV	−31.0 mV	−3.5 mV	−24.8 mV

**Figure 7:**
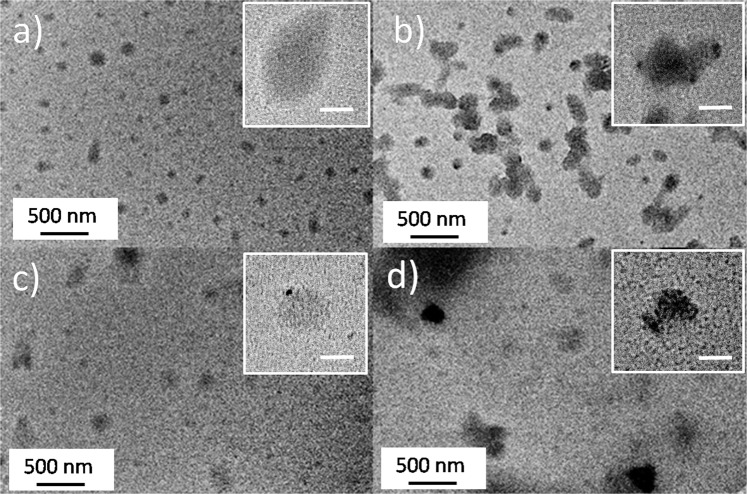
(**a**,**b**) Representative TEM images of CU2 and CU25 in ethanol; (**c**) and (**d**) CU2 and C25 modified with APTES in ethanol. The insets show the magnification of single C-dots in the different samples (scale bar = 50 nm).

C-dots have been analysed by TEM to study the morphological changes after APTES modification in ethanol. Figures 7a,b show the as prepared CU2 and CU25 samples, as a reference. In both cases, the dots are round-shaped with a large size distribution, which ranges from 80 to ≈190 nm. The TEM images, however, do not allow measuring a precise C-dots size because of a partial clusterisation of several dots in the dried state. The C-dots have been deposited on the TEM grids by casting a few droplets of samples dispersed in ethanol and the solvent evaporation results in an aggregation of C-dots.

After surface modification, the C-dots do not show specific morphological differences (Fig. 7c,d). The low contrast provided by the C-dots deposited on the TEM grids makes, however, extremely difficult to identify with precision the edge of the nanoparticles. We have also tried to magnify isolated C-dots, reported as an inset, to get a better view of the nanoparticle surface, we did not observe any evidence of a core-shell structure, eventually obtained by APTES polycondensation on the C-dots surface, in agreement with FTIR and Raman data.

### Sols and films with C-dots

**Figure 8:**
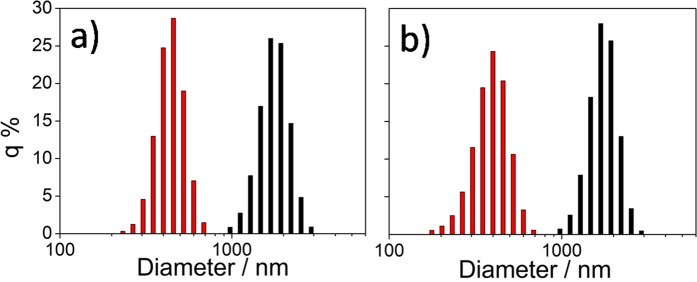
Light scattering analysis of C-dots in the precursor solution (black bars) and upon functionalization with APTES in ethanol (red bars) for CU2 (**a**) and CU25 (**b**) samples.

Mixing the as prepared C-dots with the precursor sol results in the formation of aggregates; these solutions have not been employed, therefore, for the film deposition. When the aqueous solution of APTES-functionalized C-dots has been added to the MTES-TEOS precursor sol, the mixture also resulted opaque and did not allow depositing optically transparent and homogeneous films. On the contrary, the ethanol solution of APTES-functionalized C-dots has allowed obtaining a stable sol with a high concentration of nanoparticles. We have analysed the two sols by light scattering, and the results have been compared with those of MTES-TEOS sols containing naked C-dots, previously dispersed in water and ethanol. The C-dots hydrodynamic radii in the four sols show that the naked particles (black bars in Fig. 8) have the tendency to agglomerate and form clusters with a Gaussian distribution peaking around 2 μm (Fig. 8). Upon functionalization by APTES, the clusters reduce the average size to ≈300 nm.

**Figure 9:**
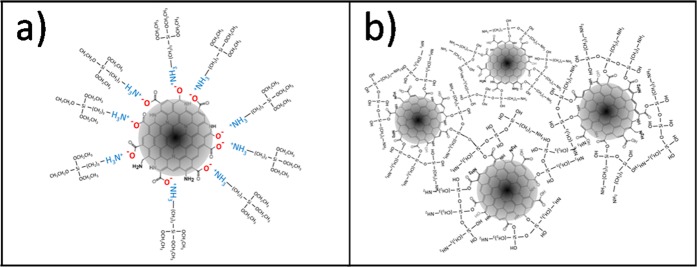
Schematics of the C-dots/APTES interactions in ethanol and water (**a**,**b**).

The FTIR and Raman characterizations (Figs. 5 and 6) have suggested that in ethanol, APTES does not react; however, as shown by light scattering, it is very effective to reduce aggregation of the particles in the precursor sol. These experimental results suggest that APTES works as a dispersing agent with the amine groups bonded to the surface of the C-dots via secondary bonds, which stabilise the particles avoiding or reducing aggregation. Considering that C-dots are generally negatively charged^32^, the interaction between APTES and C-dots surface should occur through hydrogen bonding, probably between the carboxylic or carbonyl ending groups of the nanoparticles and the amino groups of the APTES (Fig. 9a**)**. In the absence of water, which is necessary for the hydrolysis and polycondensation of the alkoxy groups, the hybrid silica precursor serves as a stabilising agent for the C-dots, avoiding clustering and precipitation through steric hindrance. On the other hand, silica thin shells form in APTES modified C-dots in water (Fig. 9b).

**Figure 10:**
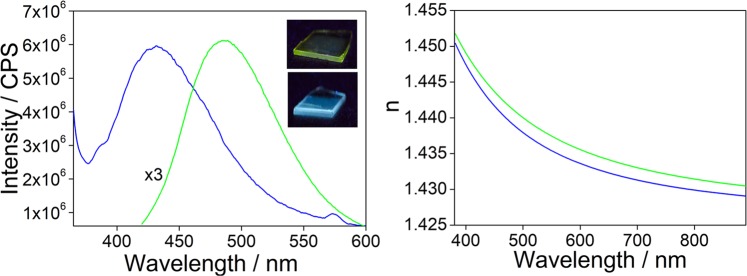
(**a**) Emission spectra of the hybrid sol-gel films containing CU2 C-dots dissolved in ethanol (blue line) or CU25 C-dots (green line) functionalized with APTES in ethanol. In the inset it is shown the picture of the films under UV lamp. (**b**) Refractive index as a function of the wavelength for CU2 (blue line) and CU25 (green line) films.

We have, therefore, used the MTES-TEOS sol containing the alcoholic solutions of APTES-functionalized C-dots to deposit hybrid organic-inorganic silica films. The films are fluorescent under UV illumination with maxima at 440 (CU2) and 490 nm (CU25) (Fig. 10). The green dots are more sensitive to the external environment than the blue dots and upon incorporation in the sol-gel matrix show a shift in the emission maximum of around 30 nm to shorter wavelengths. The films have a thickness of around 1 μm and a refractive index of 1.464 and 1.446 for CU2 and CU25, respectively.

## Conclusions

A simplified synthesis based on citric acid and urea has been designed to produce highly fluorescent blue and green C-dots with a high quantum yield, among the highest reported so far. Time-resolved photoluminescence has revealed that both blue and green emissions have a decay time of around 10 nm, the blue contribution being faster than the green. Functionalization by APTES has shown to be very effective to incorporate the C-dots into a hybrid film. In the aqueous sol, APTES reacts with water and forms a silica shell around the C-dots that avoids emission quenching, but causes the formation of large aggregates which are not suitable for film deposition. In ethanol, the hydrolysis and condensation of APTES do not occur and the organosilane stabilises the C-dots acting as a surfactant via secondary bonding of the primary amines with the surface groups. C-dots functionalized with APTES in ethanol can be used to prepare hybrid-inorganic materials via liquid phase deposition and optically transparent blue and green fluorescent films have been obtained. The present method allows achieving a full integration of C-dots chemistry with sol-gel processing.

## Methods

### Chemicals and reagents

Citric acid monohydrate (CA) (purity 99.9%) (Fluka), urea for electrophoresis (purity 98%) (Sigma), 3-(aminopropyl)triethoxysilane (APTES, 98%) (Sigma), tetraethylorthosilicate (TEOS) (Aldrich, 99% purity), methyltriethoxysilane (MTES) (Aldrich, 98% purity), ethanol (EtOH) (Fluka, >99.8%), hydrochloric acid (Sigma-Aldrich, 37% wt/wt), quinine anhydrous (Sigma, >98%), rhodamine 6 G (Sigma), sulfuric acid (Sigma, 99.9%) and water (milli-Q) were used as received without further purification. 381 mm-thick silicon wafers (Si-Mat) and silica slides (Heraeus suprasil 25 × 25 mm slides) were used as substrates for film deposition. The silicon wafers and silica slides were washed with acetone and ethanol and then dried with compressed air before film deposition.

### Synthesis of C-dots from citric acid and urea

The synthesis of C-dots has been done using two components: citric acid and urea. Two different citric acid / urea molar ratios: 1:2 and 1:25 (samples CU2 and CU25, respectively) were employed. CA and urea were dissolved in 10 mL of mQ water and heated in an open vessel using an oil bath at 190 °C for 2 h. The final product was dissolved in water or ethanol and purified in a centrifuge at 12000 rpm, for 20 min, to remove the large particles. Then the surnatant was filtered with a 0.20 µm syringe filter. Solutions with a concentration of 0.1 mg mL^−1^ and 1 mg mL^−1^ were prepared for the UV-Vis and for photoluminescence (PL) analysis, respectively. The final product obtained after 2 hours of reaction appeared as a black solid. For the functionalization with APTES, 10 mg of the final product were dissolved in 10 mL of mQ water, sonicated 5 minutes to complete the dissolution and then analyzed by UV-Vis and fluorescence spectroscopy.

### Functionalization of C-dots with APTES

The C-dots prepared with the two different syntheses were functionalized with APTES using ethanol or water as solvent. 330 µL of APTES (3.33% v/v) were added to 10 mL of C-dots in water or EtOH (1 mg mL^−1^). The solutions were reacted at 25 °C under stirring at 500 rpm in a closed vessel for 72 h; UV and PL spectra were recorded at different reaction times. The pH of the C-dots solutions in water changed from neutral to basic (pH > 10) after the addition of APTES for both CU2 and CU25.

### Precursor sol preparation

The precursor sol to be employed for film deposition was prepared by mixing 3 mL C-dots/APTES in EtOH with 1 mL EtOH, 6 mL MTES, 2 mL TEOS, 0.3 mL water and 0.2 mL HCl (2 M) in a glass vial (molar ratios, MTES:TEOS:EtOH:H_2_O:HCl = 3.4: 1.0: 7.7: 3.7: 0.1). The sol was stirred for 21 h at room temperature in a closed glass bottle. Then 200  mL of milli-Q water were added to the hybrid sol (MTES–TEOS) and the solution was left to react under stirring for 2 h before deposition.

### Preparation of hybrid organic-inorganic films

The hybrid films were deposited by dip-coating onto silicon wafers and silica slides with a withdrawal rate of 15 cm min^−1^. The deposition was performed at 25 °C and 24% relative humidity; the as deposited films were then placed in an oven at 60 °C. Transparency of the hybrid films was measured with a Nicolet Evolution 300 UV–Vis spectrophotometer in the range 200–600 nm with a bandwidth of 1.5 nm. A clean quartz slide was used as a background reference. PL measurements were collected on silica slides with excitation at 350  and 400 nm for blue and green emitting films, respectively.

### Characterization

UV-Vis measurements were performed in absorbance, using a Nicolet Evolution 300 spectrophotometer from 200 to 600 nm, on C-dots solutions at concentration 0.1 mg mL^−1^. Fluorescence spectroscopic measurements of C-dots solubilized in water and EtOH and on films deposited on silica slides, were performed using a Horiba Jobin Yvon FluoroMax-3 spectrofluorometer; three-dimensional mapping was obtained with a 450 W xenon lamp as the excitation source. Three-dimensional maps were collected with an excitation range of 200–600 nm and an emission range of 200–600 nm with a 5 nm slit for excitation and emission.

Fourier-transform infrared (FTIR) spectroscopy analysis was performed with a Bruker infrared Vertex 70 interferometer. The spectra were recorded in absorbance mode between 4000 and 400 cm^−1^ by averaging 256 scans with 4 cm^−1^ of resolution. 2 mg of solid were used to prepare KBr pellets for measurements in transmission mode. Attenuated total reflectance (ATR) mode was used to direclty analyze the solids or the evaporating solutions. For the films a silicon wafer was used as the background; the data were analyzed with OPUS 7.0 and ORIGIN Pro 8 software.

Micro Raman scattering measurements were carried out in back scattering geometry with the 1064 nm line of an Nd:YAG laser. Measurements were performed in air at room temperature with a B&W TEK (Newark-USA) i-Raman Ex integrated system with a resolution of about 8 cm^−1^.

As previously reported, a Wollam-spectroscopic ellipsometer with fixed angle geometry was used to estimate the thickness and refractive index dispersions of the hybrid films deposited on silicon substrates, by fitting the experimental data with a model for transparent films on Si substrates. The fit showed an average mean square error always lower than 6.5^33^.

Time resolved photoluminescence (TR-PL) measurements were recorded by exciting the samples with 200 fs long pulses delivered by an optical parametric amplifier (Light Conversion TOPAS-C) pumped by a regenerative Ti:sapphire amplifier (Coherent Libra-HE). The repetition frequency was 1 kHz and the PL signal was recovered by a streak camera (Hamamatsu C10910) equipped with a grating spectrometer (Princeton Instruments Acton SpectraPro SP-2300). All the measurements were collected in the front face configuration to reduce inner filter effects. Proper emission filters were applied to remove the reflected contribution of the excitation light, as reported in previous studies^34^.

Zeta potential (z) and light scattering were measured on a Zetasizer Nano ZSP (Malvern Instruments) operating in backscatter configuration (θ = 173°) at laser wavelength of λ = 633 nm. Samples were syringe filtered and transferred into a capillary folded elecrophoretic cell for analysis at 25 °C.

Quantum Yield (QY) of the C-dots were measured by a relative method; quinine sulfate (QY = 55% in 0.1 M H_2_SO_4_) was used as the reference for the 400–480 nm emission range (for CA: Urea = 1: 2), rhodamine 6 G (QY = 95% in ethanol) for the 480–560 nm emission range (for CA: Urea = 1: 25). The QY was then calculated using Eq. (1) from ref. ^22^.

1 $$\phi ={\phi }^{{\prime} }\frac{{A}^{{\prime} }}{{I}^{{\prime} }}\frac{{\rm{I}}}{{\rm{A}}}\frac{{{\rm{n}}}^{2}}{{{{\rm{n}}}^{{\prime} }}^{2}}$$

where ϕ is the QY of the testing sample, I is the testing sample’s integrated emission intensity, n is the refractive index (1.33 for water and 1.36 for ethanol), and A is the optical density. Φ′, A′, I′ and n′ are values of the referenced fluorescence dyes of known QYs. To obtain the QY values for CU2 we have used the absorbance at 365 nm and an excitation at the same wavelength; the emission spectra have been integrated in the 380–600 nm range, using quinine as reference. The QY for CU25 has been, instead, calculated using the absorbance and excitation at 420 nm and integrating the emission between 430 and 700 nm.

To obtain reliable results, the C-dots and reference dyes solutions were diluted to have an optical absorbance between 0 and 0.1. The data were analyzed using ORIGIN software. QYs were calculated by comparison of the integrated PL intensity vs absorbance curves (refractive index, n, was also considered).

Absolute photoluminescence quantum yield (AQY) measurements have been performed using the quanta-ϕ (HORIBA) integrating sphere accessory, attached to the “NanoLog” spectrofluorometer. Water or ethanol were used as a blank ref. ^34^.

Transmission electron microscopy (TEM) images were obtained by using a FEI TECNAI 200 microscope working with a field emission electron gun operating at 200 kV. Sample preparation was done by dispersing the nanoparticles in ethanol by ultrasonication and then dropping them onto a carbon-coated copper grid and drying them for observations^35^.

## Supplementary information

Supplementary Information.

## References

[1] Zholobak NM (2016). Facile fabrication of luminescent organic dots by thermolysis of citric acid in urea melt, and their use for cell staining and polyelectrolyte microcapsule labelling. Beilstein J. Nanotechnol..

[2] Ding H, Wei JS, Zhong N, Gao QY, Xiong HM (2017). Highly Efficient Red-Emitting Carbon Dots with Gram-Scale Yield for Bioimaging. Langmuir.

[3] Ludmerczki R (2019). Carbon dots from citric acid and its intermediates formed by thermal decomposition. Chem. Eur. J..

[4] Khan WU (2017). High quantum yield green-emitting carbon dots for Fe(III) detection, biocompatible fluorescent ink and cellular imaging. Sci. Rep..

[5] Schneider J (2017). Molecular fluorescence in citric acid-based carbon dots. J. Phys. Chem. C.

[6] Lin Y (2015). Tunable Fluorescent Silica-Coated Carbon Dots: A Synergistic Effect for Enhancing the Fluorescence Sensing of Extracellular Cu 2+ in Rat Brain. ACS Appl. Mater. Interfaces.

[7] Panniello A (2018). Luminescent Oil-Soluble Carbon Dots toward White Light Emission: A Spectroscopic Study. J. Phys. Chem. C.

[8] Suzuki K (2017). Design of Carbon Dots Photoluminescence through Organo-Functional Silane Grafting for Solid-State Emitting Devices. Sci. Rep..

[9] Bhattacharya D, Mishra MK, De G (2017). Carbon Dots from a Single Source Exhibiting Tunable Luminescent Colors through the Modification of Surface Functional Groups in ORMOSIL Films. J. Phys. Chem. C.

[10] Vassilakopoulou A, Georgakilas V, Koutselas I (2017). Encapsulation and protection of carbon dots within MCM-41 material. J. Sol-Gel Sci. Technol..

[11] Cheng J, Wang C-F, Zhang Y, Yang S, Chen S (2016). Zinc ion-doped carbon dots with strong yellow photoluminescence. RSC Adv..

[12] Simões EFC, Leitão JMM, da Silva JCGE (2016). Carbon dots prepared from citric acid and urea as fluorescent probes for hypochlorite and peroxynitrite. Microchim. Acta.

[13] Kasprzyk W (2018). Luminescence phenomena of carbon dots derived from citric acid and urea-a molecular insight. Nanoscale.

[14] Sciortino A (2018). β-C3N4 Nanocrystals: Carbon Dots with Extraordinary Morphological, Structural, and Optical Homogeneity. Chem. Mater..

[15] Lai CW, Hsiao YH, Peng YK, Chou PT (2012). Facile synthesis of highly emissive carbon dots from pyrolysis of glycerol; Gram scale production of carbon dots/mSiO_2_ for cell imaging and drug release. J. Mater. Chem..

[16] Peng Y, Zhou X, Zheng N, Wang L, Zhou X (2017). Strongly tricolor-emitting carbon dots synthesized by a combined aging-annealing route and their bio-application. RSC Adv..

[17] Chandra S, Beaune G, Shirahata N, Winnik FM (2017). A one-pot synthesis of water soluble highly fluorescent silica nanoparticles. J. Mater. Chem. B.

[18] Liu C (2016). Fluorescence-Converging Carbon Nanodots-Hybridized Silica Nanosphere. Small.

[19] Malfatti L, Innocenzi P (2018). Sol-Gel Chemistry for Carbon Dots. Chem. Rec..

[20] Suzuki K (2015). Energy transfer induced by carbon quantum dots in porous zinc oxide nanocomposite films. J. Phys. Chem. C.

[21] Innocenzi P, Malfatti L, Carboni D (2015). Graphene and carbon nanodots in mesoporous materials: an interactive platform for functional applications. Nanoscale.

[22] Carbonaro CM (2018). Carbon Dots in Water and Mesoporous Matrix: Chasing the Origin of their Photoluminescence. J. Phys. Chem. C.

[23] Jiang K (2015). Red, green, and blue luminescence by carbon dots: Full-color emission tuning and multicolor cellular imaging. Angew. Chemie - Int. Ed..

[24] Würth C, Grabolle M, Pauli J, Spieles M, Resch-Genger U (2013). Relative and absolute determination of fluorescence quantum yields of transparent samples. Nat. Protoc..

[25] Hiraoui M (2011). Spectroscopy studies of functionalized oxidized porous silicon surface for biosensing applications. Mater. Chem. Phys..

[26] Jang M, Cho I, Callahan P (1997). Adsorption of Globular Proteins to Vaccine Adjuvants. Journal of Biochemistry and Molecular Biology.

[27] Ruan C, Wang W, Gu B (2006). Detection of alkaline phosphatase using surface-enhanced raman spectroscopy. Anal. Chem..

[28] Pryce RS, Hench LL (2004). Tailoring of bioactive glasses for the release of nitric oxide as an osteogenic stimulus. J. Mater. Chem..

[29] Shih PTK, Koenig JL (1975). Raman studies of silane coupling agents. Mater. Sci. Eng..

[30] King S (1967). Ring Configurations in a Random Network Model of Vitreous Silica. Nature.

[31] Galeener FL, Barrio RA, Martinez E, Elliot RJ (1984). Vibrational Decoupling of Rings in Amorphous Solids. Phys. Rev. Lett..

[32] Sciortino A, Cannizzo A, Messina F (2018). Carbon Nanodots: A Review—From the Current Understanding of the Fundamental Photophysics to the Full Control of the Optical Response. C.

[33] Jang Y (2016). Hard X-rays for processing of hybrid organic-inorganic thick films. J. Sync. Rad..

[34] Ren J (2019). Boron oxynitride two-colour fluorescent dots and their incorporation in a hybrid organic-inorganic film. J. Colloid Interface Sci..

[35] Carboni D, Lasio B, Malfatti L, Innocenzi P (2016). Magnetic core-shell nanoparticles coated with a molecularly imprinted organogel for organophosphate hydrolysis. J. Sol-Gel Sci. Technol..

