# Supplementary material for: Integrating sol-gel and carbon dots chemistry for the fabrication of fluorescent hybrid organic-inorganic films
Source: Sci Rep. 2020 Mar 16;10:4770. doi: 10.1038/s41598-020-61517-x (PMC7075866; doi:10.1038/s41598-020-61517-x)
Supplement: Supplementary file 1 — Supplementary Information. [file 41598_2020_61517_MOESM1_ESM.docx]

**Supplementary Information**

***Integrating sol-gel and carbon dots chemistry for the fabrication of fluorescent hybrid organic-inorganic films***

### Stefania Mura,^1^ Róbert Ludmerczki^1^, Luigi Stagi^1^, Sebastiano Garroni^2^, Carlo Maria Carbonaro^3^, Pier Carlo Ricci^3^, [Maria Francesca Casula](about:blank)^4^, Luca Malfatti^1^, Plinio Innocenzi^1*^.

^1^Laboratorio di Scienza dei Materiali e Nanotecnologie, CR-INSTM, Dipartimento di Chimica e Farmacia, Università di Sassari,  Via Vienna 2, 07100 Sassari. Italy.

^2^Dipartimento di Chimica e Farmacia, Università di Sassari,  Via Vienna 2, 07100 Sassari. Italy.

*^3^Department of Physics, University of Cagliari, Campus of Monserrato, sp n.8, km 0.700, 09042 Monserrato. Italy*

*^4^DIMCM-Department of Mechanical, Chemical, and Materials Engineering INSTM and University of Cagliari Via Marengo 2, I 09123 Cagliari, ITALY*

**Correspondence to plinio@uniss.it*

**Fig. S1** a) UV-vis absorption spectra of citrazinic acid at a concentration of 0.1 mg mL^-1^ in water and b) 3D spectrum collected at a concentration 1 mg L^-1^.


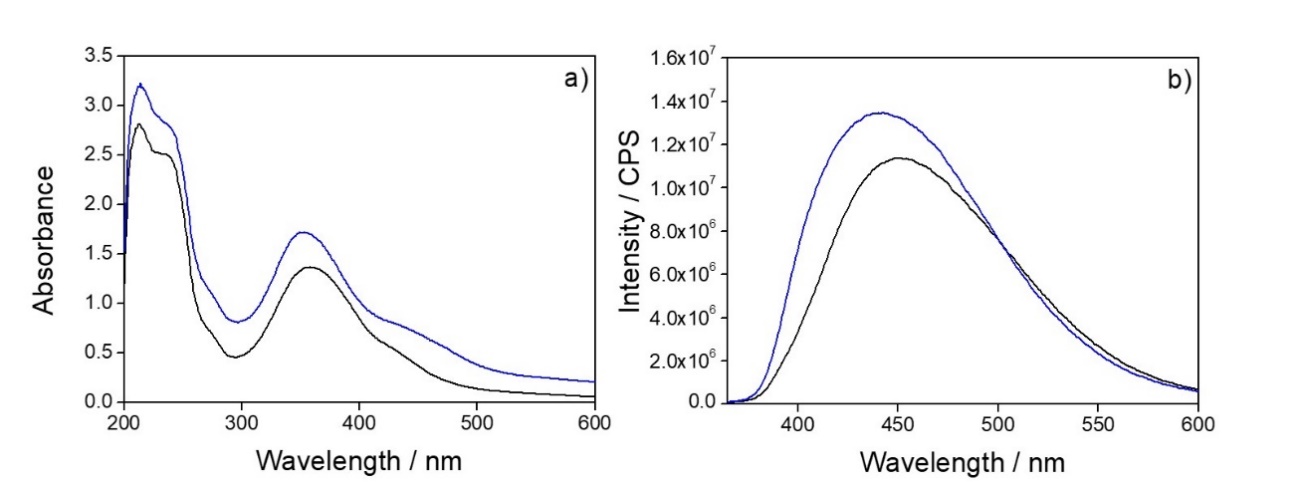


**Fig. S2** a) UV-vis absorption spectra of CU2 C-dots (concentration 0.1 mg mL^-1^) before (black line) and after 24 hours of reaction with APTES (blue line); b) emission spectra of CU2 in EtOH at a concentration 1 mg L^-1^ excited at 350 nm before (black line) and after 24 hours of reaction with APTES (blue line).


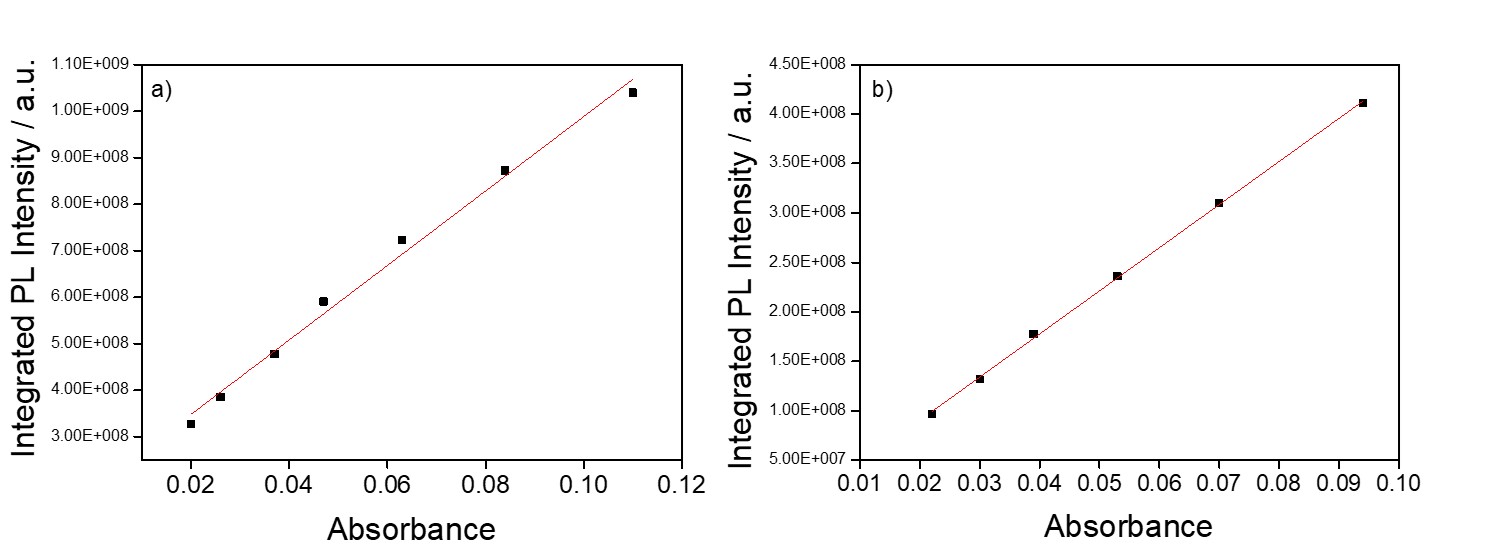


**Fig. S3** Plots of integrated PL intensity of a) quinine sulfate (reference dye) in H_2_SO_4_ 0.1M and b) CU2 C-dots dissolved in in H_2_SO_4_ 0.1M, as a function of optical absorbance at 365 nm.


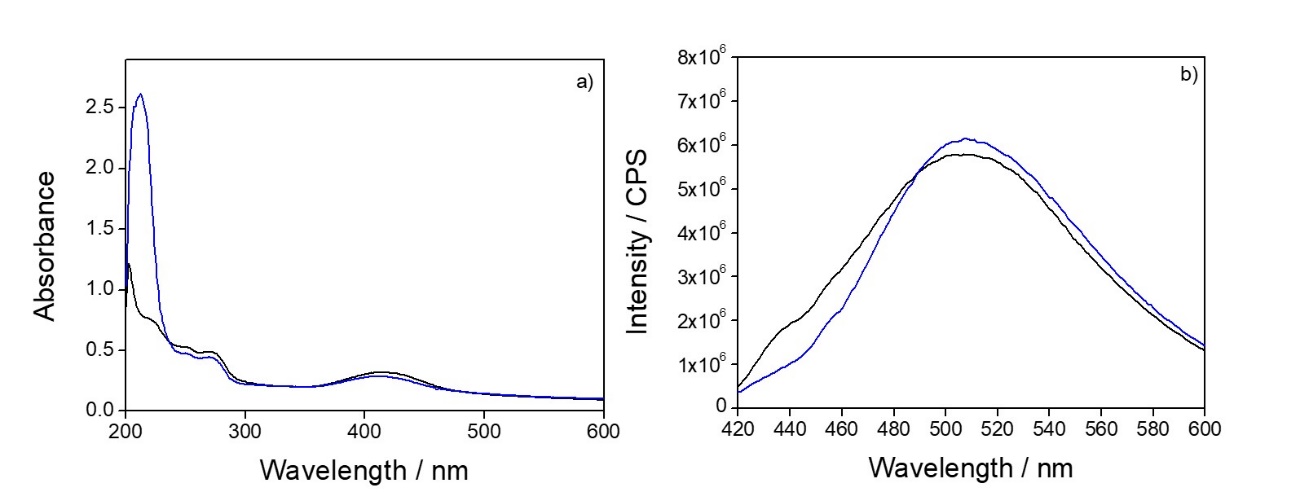


**Fig. S4** a) UV-vis absorption spectra of alcoholic solutions of CA:Urea CDs 1:25 (concentration 0.1 mg mL^-1^) before (black line) and after (blue line) the functionalization with APTES in EtOH. b) Emission spectra of CDs in EtOH (concentration 1 mg L^-1^) (λ_ex_ = 400 nm) before (black line) and after the functionalization with APTES (blue line).


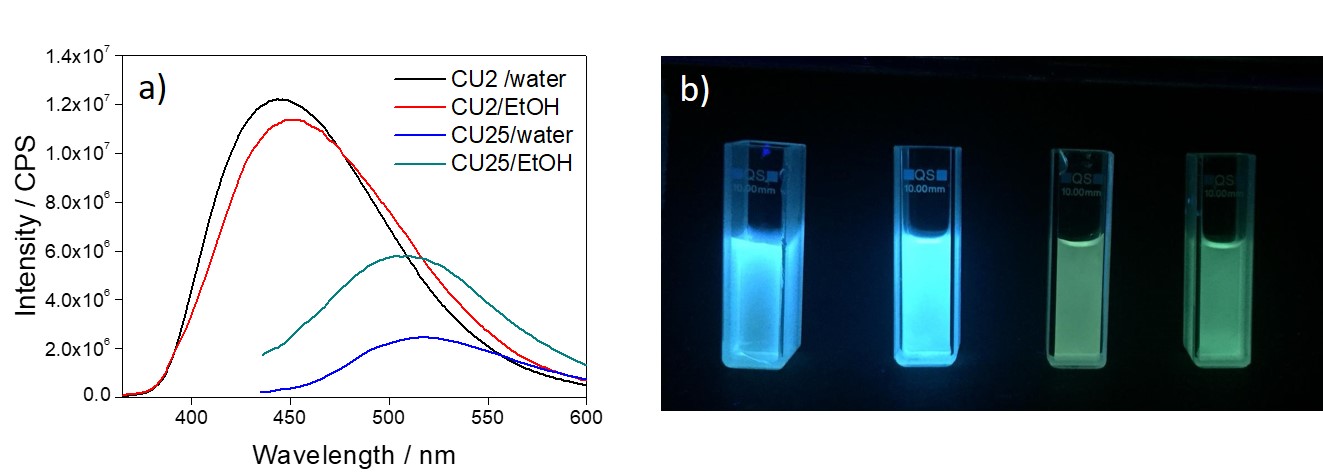


**Fig. S5** a) Emission spectra of CU2 C-dots (1 mg L^-1^ concentration, λ_ex_ = 350 nm) and CU25 C-dots (1 mg L^-1^ concentration, λ_ex_ = 400 nm) in water and EtOH b) Digital images of CU2 C-dots in water and EtOH (blue emitting) and CU25 C-dots in water and EtOH (green emitting), respectively from the left to the right .


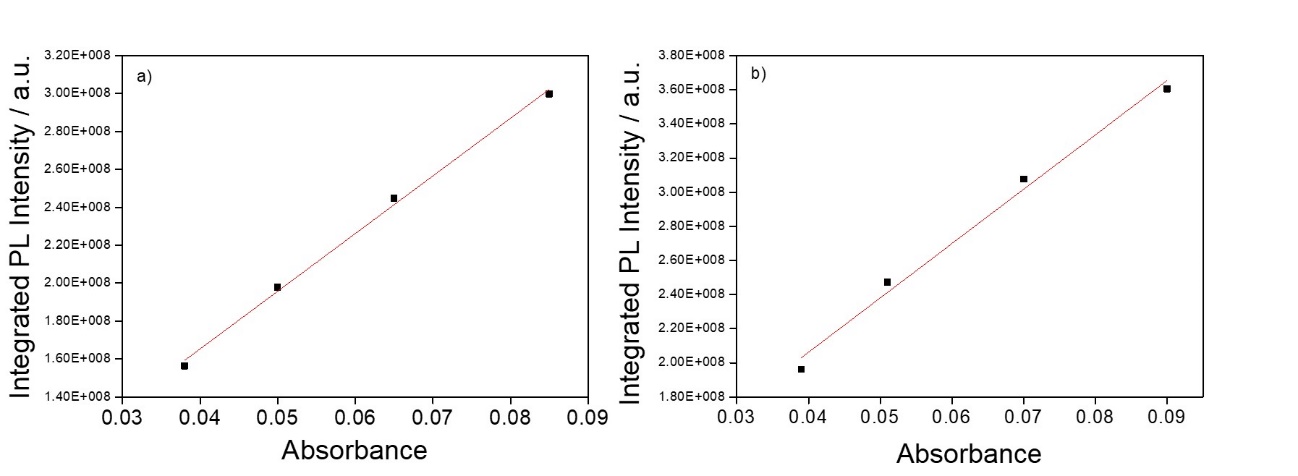


**Fig. S6** Plots of integrated PL intensity of Rh6G in EtOH (reference dye) a) with CA:Urea 1:25 CDs b) as a function of optical absorbance at 420 nm.

**CU2 in water CU2 in ethanol**


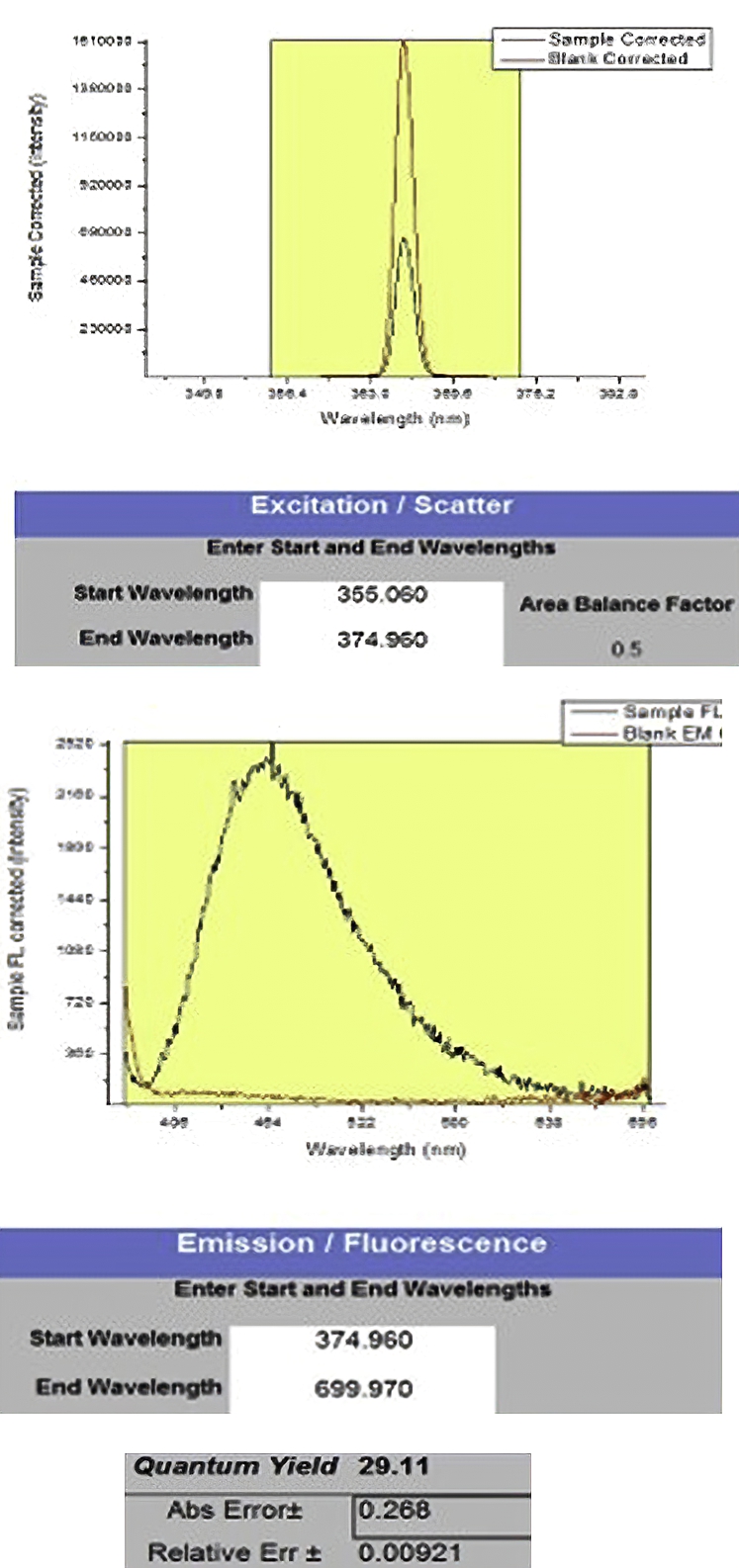

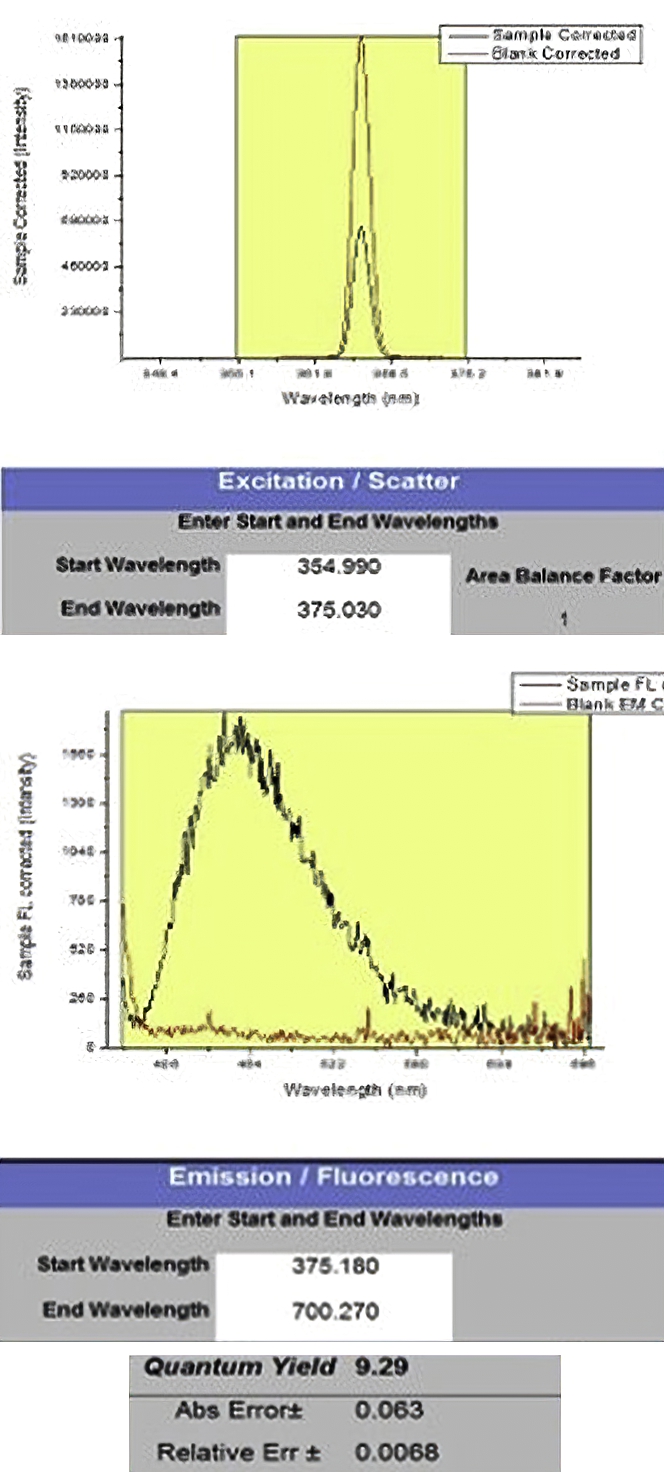


**CU25 in water CU25 in ethanol**


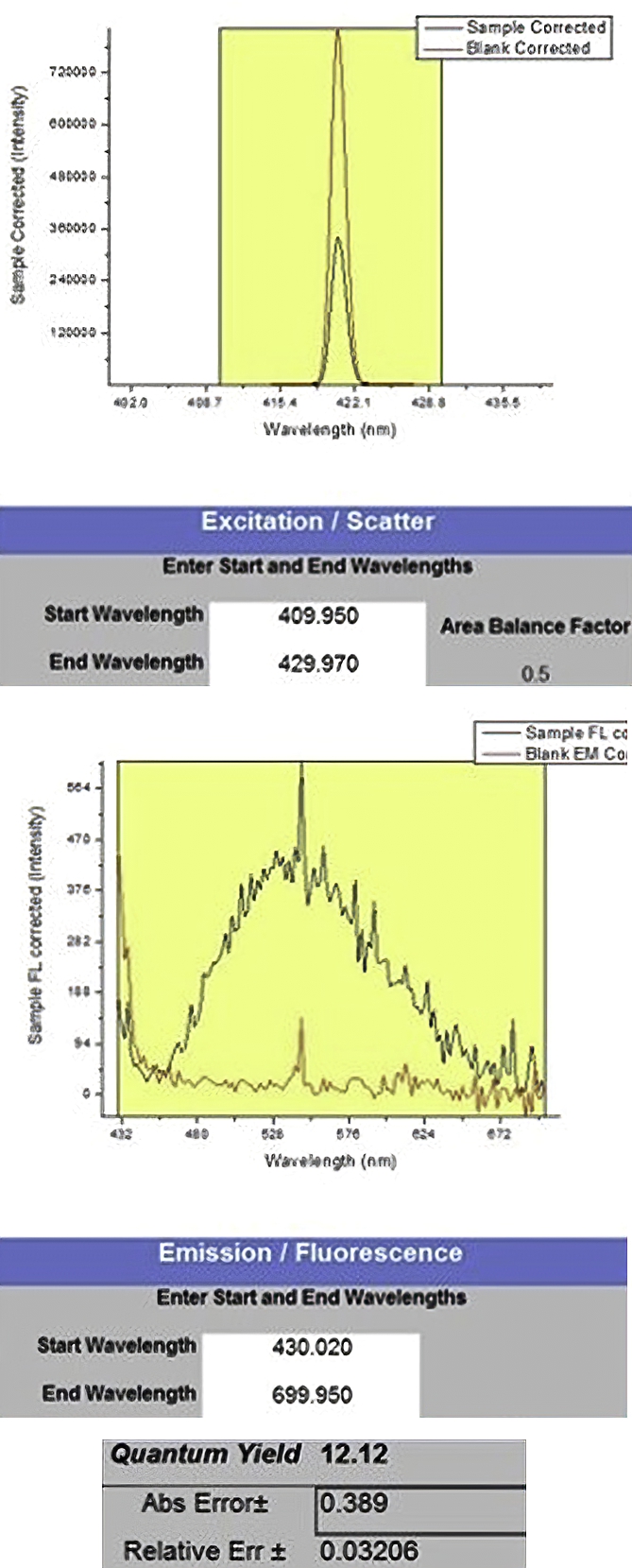

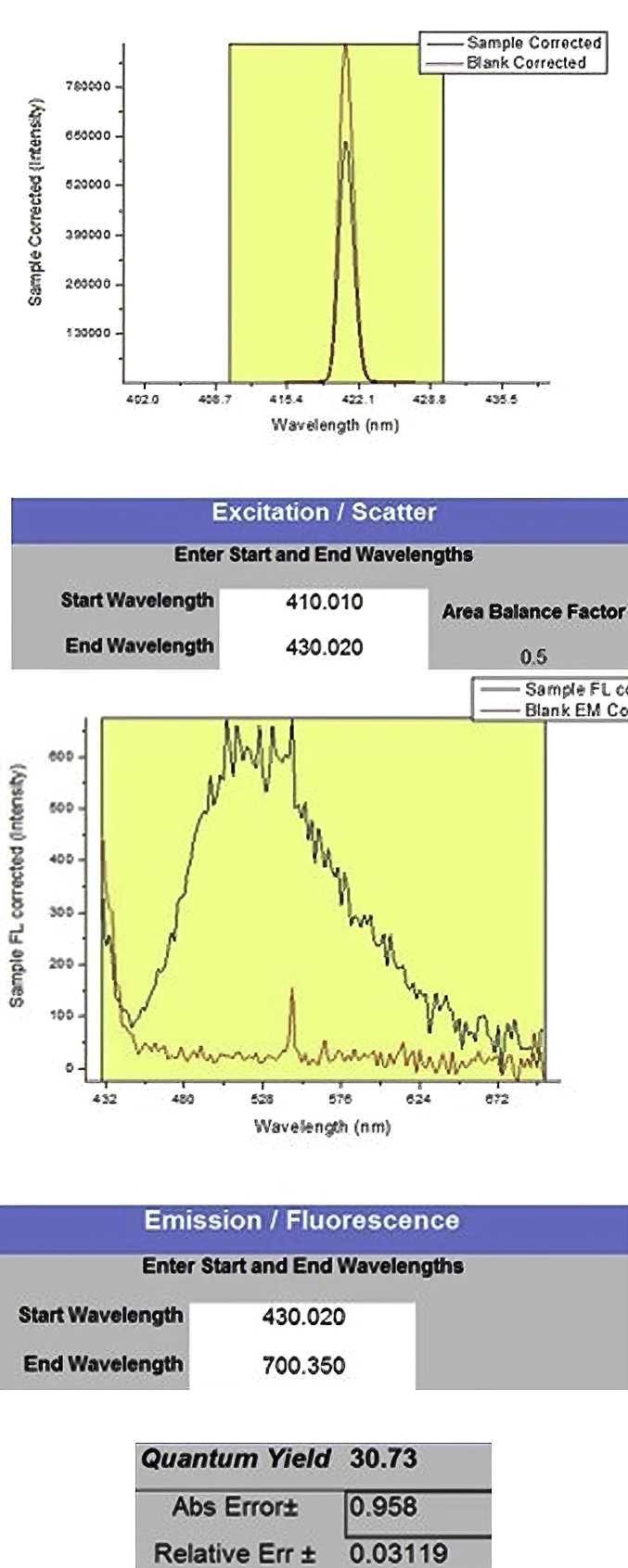


**Fig. S7** Photoluminescence quantum yield (PLQY) measurements of CU2 and CU25 C-dots using water or ethanol as a blank.

**Tab. S1** QY calculations for CU2 and CU25 CDs using respectively quinine and Rh6G as reference.

|  | **CU2 CDs** | **Quinine**  ** ex. 365 nm** | **CU25 CDs** | **Rhodamine 6G ex 420 nm** |
| --- | --- | --- | --- | --- |
| **Slope** | 4.36093E9 | 7.99509E9 | 3.1857E9 | 3.04115E9 |
| **QY%** | 30% | 55% | 99.5% | 95% |

**Tab. S2** Decay time profile of CU2 and CU25 C-dots before and after modification with APTES in water and in ethanol excited at 350 nm and 400 nm respectively.

| **SAMPLES** | **λex= 350 λem=450** | **λex= 400 λem=510** |
| --- | --- | --- |
| **CU2/water** | 10.0 ns |  |
| **CU2/EtOH** | 8.7 ns |  |
| **CU25/water** |  | 5.6 ns |
| **CU25/EtOH** |  | 10.0 ns |
| **CU2/water+APTES** | 10.0 ns |  |
| **CU2/EtOH+APTES** | 10.0 ns |  |
| **CU25/water+APTES** | 8.7 ns |  |
| **CU25/EtOH+APTES** |  | 11.6 ns |


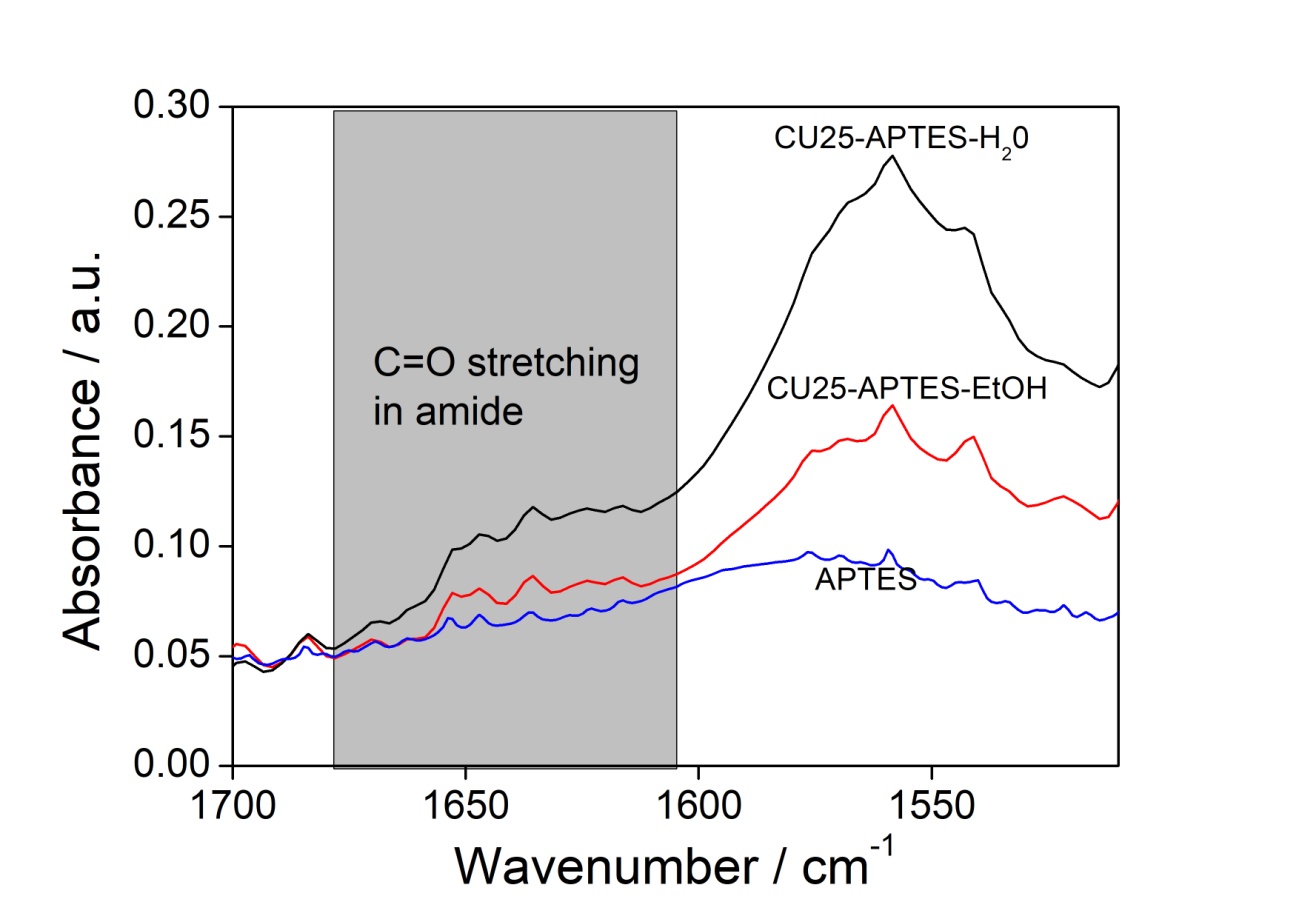


**Fig. S8** FTIR absorption spectra in the 1700 – 1500 cm^-1^ range. CU25 C-dots modified with APTES in water (black line) or in ethanol (red line); the APTES spectrum is shown as a reference (blue line)
